# Yttrium-90 Induces an Effector Memory Response with Neoantigen Clonotype Expansion: Implications for Immunotherapy

**DOI:** 10.1158/2767-9764.CRC-24-0228

**Published:** 2024-08-19

**Authors:** Kelley Núñez, Tyler Sandow, Juan Gimenez, Mina Hibino, Ari Cohen, Paul Thevenot

**Affiliations:** 1 Institute of Translational Research, Ochsner Health System, New Orleans, Louisiana.; 2 Interventional Radiology, Ochsner Health System, New Orleans, Louisiana.; 3 Multi-Organ Transplant Institute, Ochsner Health System, New Orleans, Louisiana.; 4 Faculty of Medicine, University of Queensland, Brisbane, Australia.

## Abstract

Yttrium-90 (^90^Y) transarterial radioembolization can safely and effectively treat hepatocellular carcinoma (HCC). Clinical trials combining ^90^Y with immunotherapy are aimed at improving treatment response rates. The impact of transient ^90^Y-induced lymphopenia on T-cell homeostasis and functional dynamics is unknown. Paired blood specimens were collected prior to first-cycle ^90^Y and at imaging follow-up in patients with HCC Barcelona Clinic Liver Cancer stages A–B. Flow cytometry and T-cell receptor (TCR) sequencing were used to monitor changes in T-cell subsets and TCR repertoire following ^90^Y. Objective response (OR) rates were determined using modified RECIST and defined as either OR or nonobjective response. Time-to-progression (TTP) was defined as progression to Barcelona Clinic Liver Cancer stage C within 6 months following ^90^Y. ^90^Y induced shifts in both CD4^+^ (*P* = 0.049) and CD8^+^ (*P* < 0.001) toward an effector memory T-cell response independent of treatment response rate. Nonresponders to ^90^Y were characterized by a sustained elevation in both naïve CD4^+^ cells (*P* = 0.019) and programmed cell death protein 1 expression in CD8^+^ cells (*P* = 0.003). Paired analysis of the TCR repertoire revealed a variable induction of neoantigen clonotypes and expansion of existing clonotypes independent of ^90^Y response. In patients with an OR, changes in TCR clonality did not influence TTP. However, polyclonal profiles in patients without an OR were associated with shorter TTP (*P* = 0.005; HR, 10.8) and 75% disease progression rates 6 months following treatment. ^90^Y induces a population shift from central to effector memory accompanied by neoantigen T-cell responses independent of treatment response rate. Monoclonal shifts in the post-^90^Y T-cell repertoire had superior overall TTP and improved TTP in patients with a first-cycle nonobjective response.

**Significance:**

^90^Y can safely treat HCC; however, it causes transient lymphopenia. In this article, ^90^Y stimulates a peripheral effector memory response independent of initial treatment response. TCR sequencing revealed that polyclonal profiles in patients without an OR to treatment were associated with rapid progression rates 6 months after ^90^Y.

## Introduction

Hepatocellular carcinoma (HCC) represents the most common type of liver cancer and ranks fourth worldwide for cancer-related deaths (1). HCC incidence rates continue to increase because of improved surveillance driven by the primary risk factors of viral hepatitis and steatotic liver disease (SLD; 2, 3). Liver transplantation remains the only curative treatment option for patients with HCC in the setting of cirrhosis. Liver-directed therapies have been used in early- to intermediate-stage disease [Barcelona Clinic Liver Cancer (BCLC) stages A–B] as a strategy to definitively treat or bridging/downstage to liver transplantation and include microwave ablation, transarterial chemoembolization, and transarterial radioembolization.

In solitary HCC (BCLC-A), transarterial radioembolization (TARE) using yttrium-90 (^90^Y) achieves excellent treatment response rates (4, 5). Despite excellent first-cycle target response rates, 12% to 16% of patients still experience disease progression through progression of the treated lesion or the development of new lesions within the liver (4, 5). Immune checkpoint inhibitors (ICI) are established as the first-line therapy for advanced stage (BCLC-C), but they represent only a modest overall improvement in the response rate compared with tyrosine kinase inhibitors with only ∼20% of patients responding to therapy (6, 7). Currently, several clinical trials are underway combining ^90^Y with ICI for unresectable HCC (NCT03099564, NCT06040099, NCT03033446, NCT04605731, and NCT05063565) in hopes of improving overall response rates.

Although optimal patient selection for ICI remains challenging, a deeper understanding of how ^90^Y affects the immune system is required to fully appreciate the impacts of combining ^90^Y with ICI to identify the responder population. Our recent work demonstrated that ^90^Y-induced lymphopenia was associated with a lung shunt fraction and did not impact treatment response or disease progression rates (8). Our study also showed that patients with elevations in programmed cell death protein 1 (PD1) on peripheral CD8^+^ T cells following ^90^Y were at increased risk of progression. Other studies have suggested an immunomodulatory role of ^90^Y for both tumor infiltrative and peripheral T cells, but with some notable limitations (9, 10). These studies utilized T cells that were polyclonally (antigen-independent) reactivated *in vitro*, potentially obscuring their functional status at the time of isolation. Additionally, the patient population consisted of ^90^Y as a bridge to resection in patients with intermediate–advanced HCC (BCLC-B and -C) in the absence of cirrhosis and therefore negating the immunomodulatory role of underlying cirrhosis. Although the available literature supports a ^90^Y-dependent influence on T-cell dynamics, the specific T-cell subsets implicated as well as their responsiveness to tumor-specific antigens and effector potential remain unknown. T-cell receptor (TCR) repertoire dynamics after ^90^Y represent a critical first step to characterizing these responses, but they have yet to be explored in the literature. TCR repertoires offer insights into the adaptive immune response and monitor treatment-induced T-cell dynamics (11).

To address these gaps, we performed paired T-cell immunophenotyping and TCR sequencing on peripheral T cells from patients with BCLC A–B HCC before and after undergoing first-cycle ^90^Y. This allowed for an in-depth analysis of ^90^Y-induced changes to peripheral T cells and the ability to address whether ^90^Y causes an immunologic change within the periphery. Understanding the immunologic landscape prior to treatment and how ^90^Y impacts this landscape could provide insights into which patients may benefit from combinational therapy.

## Materials and Methods

### Study cohort and clinical variables

A prospective, single-center study was conducted and approved by the Ochsner Health System Institutional Review Board (protocol # 2016.131.B) and was in accordance with the ethical guidelines set forth by the 1975 Declaration of Helsinki. Written informed consent was obtained from each participant with enrollment dates from November 2017 to December 2022. Study inclusion criteria were as follows: (i) confirmed HCC diagnosis by biopsy or triple-phase imaging according to the Liver Imaging Reporting and Data System criteria (v2018 American College of Radiology), (ii) unresectable HCC, (iii) BCLC stages A and B, (iv) ages ≥18 years, and (v) scheduled to undergo ^90^Y-TARE as first-cycle treatment.

Clinical variables were extracted from the electronic medical record following HCC diagnosis prior to treatment and at clinical follow-up (<6 months post-^90^Y). Extracted variables were as follows: general demographics, cirrhosis history, model of end-stage liver disease scores, complete blood counts, and complete metabolic liver panels. Decompensation history at HCC diagnosis was defined as the presence of hepatic encephalopathy, ascites, or bleeding esophageal varices that required surgical or pharmacologic intervention. HCC burden was obtained from triple-phase CT/MRI studies following consensus review by the institution’s multidisciplinary HCC board.

### ^90^Y-TARE treatment protocol and assessment of treatment response

All ^90^Y-TARE procedures were performed at a single interventional oncology site (Ochsner Health System). HCC diagnoses were determined following review by a multidisciplinary HCC board consisting of interventional radiologists, oncologists, hepatologists, and liver transplant surgeons. The institutional treatment algorithm for first-cycle treatment with ^90^Y required unresectable HCC with an index tumor ≥3 or <3 cm and not amendable to microwave ablation. Inclusion criteria for ^90^Y as first-cycle treatment for HCC included (i) Child–Pugh A–B, (ii) absence of portal vein thrombus, (iii) no extrahepatic metastasis, (iv) total bilirubin <4 mg/dL, (v) serum creatinine concentration <1.5 mg/dL, and (vi) absence of gross ascites. The ^90^Y-TARE procedure was performed as a two-phase treatment that included a mapping angiogram followed by deployment of the ^90^Y microsphere infusion (TheraSphere, Boston Scientific). Vascular evaluation of the celiac, superior mesenteric, proper hepatic, and all feeding hepatic arteries to the tumor was performed during the mapping angiography. All patients received contrast-enhanced cone beam CT to confirm both coverage of tumor angiosome and to calculate the perfused volume for TARE. During mapping angiogram, technetium 99m–labeled macroaggregated albumin was used to calculate the lung shunt fraction. Medical internal radiation dose was used and incorporated perfused volumes and lung shunt fraction to calculate the desired dose to the perfused location. During radioembolization, all vessels feeding to the areas of tumor were treated with target radiation doses >200 Gy and delivered via segmental or subsegmental. Pre- and post-^90^Y dosimetry calculations were performed using Mirada DBx Build 1.2.0 and Simplicit^90^Y using pre-^90^Y CT/MRI imaging with either pre- or post-^90^Y single-photon emission computed tomography nuclear medicine and CT scans. Procedures prior to 2020 did not have post-^90^Y single-photon emission computed tomography nuclear medicine and CT scans performed. All procedures after 2019 had tumor-absorbed dose calculated from Simplicit^90^Y. All ^90^Y procedures were performed by board-certified radiologists according to institution’s standard protocol.

Routine follow-up imaging post-^90^Y was performed using either MRI or CT. First-cycle response to ^90^Y was evaluated using the modified RECIST (mRECIST) for HCC (12) by a board-certified interventional radiologist with >8 years of experience. The target objective response rate (tORR) was evaluated as a response to the initial lesion treated during first-cycle ^90^Y and defined as either an objective response (OR) corresponding to complete and partial responses of the mRECIST or nonobjective response (NOR) corresponding to stable disease and disease progression of the mRECIST. The overall objective response rate (oORR) was evaluated as a response to the treated lesion, included the development of new lesions in adjacent liver segments, and was defined as either OR or NOR.

### Study endpoints

The primary endpoint was rapid time-to-progression (TTP) defined as BCLC-A or B to BCLC-C within 6 months following first-cycle ^90^Y as determined by the institution’s multidisciplinary HCC board. The secondary endpoints were tORR and oORR.

### Blood collections and peripheral blood mononuclear cell isolation

Peripheral blood was collected immediately prior to first-cycle ^90^Y, and a second blood collection occurred during routine post-^90^Y imaging. All blood was collected in sodium citrate cell preparation tubes (BD Biosciences) and processed within 4 hours of collection. Peripheral blood mononuclear cells (PBMC) were isolated by Ficoll gradient and then cryopreserved in 10% DMSO (Sigma-Aldrich) and 90% FBS (R&D Systems).

### Flow cytometry

Multiple panels were used to stain for T-cell phenotypes from unstimulated PBMCs and were grouped based on markers for the following: (i) memory, (ii) senescence, (iii) exhaustion, and (iv) PD1. The memory panel consisted of the following antibodies (Life Technologies): anti–CD28 FITC (clone CD28.2), anti–CD95 PE (clone DX2), anti–CD45RO PerCP (clone UCHL1), anti–CD4 PE-Cy7 (clone RPA-T4), anti–CCR7 APC (clone 3D12), and anti–CD8 APC-Cy7 (clone RFT-8). The senescent panel contained the following antibodies: anti–CD28 FITC (clone CD28.2), anti–CD57 PE (clone TBO1), anti–CD56 PerCP (clone MEM-188), anti–CD4 PE-Cy7 (clone RPA-T4), anti–KLRG1 APC (clone 13F12F2), and anti–CD8 APC-Cy7 (clone RFT-8). The exhaustion panel consisted of the following antibodies: anti–CTLA4 FITC (clone 14D3), anti–CD56 PerCP (clone MEM-188), anti–CD4 PE-Cy7 (clone RPA-T4), anti–LAG3 APC (clone 3DS223H), and anti–CD8 APC-Cy7 (clone RFT-8). The final panel for PD1 expression contained the following antibodies: anti–CD3 FITC (clone UCHT1), anti–CD56 PE (clone CMSSB), anti–CD8 PerCP-Cy5.5 (clone RPA-T8), anti–PD1 APC (clone J105), anti–CD45 Alexa Fluor 700 (clone 2D1), and anti–CD4 APC-Alexa Fluor 780 (clone RPA-T4).

### TCR sequencing

Genomic DNA was isolated from cryopreserved PBMCs using QIAamp DNA Blood Midi kit (Qiagen). Extracted DNA was quantified using Qubit 4 (Thermo Fisher Scientific). TCR β-chain sequencing was performed using the immunoSEQ platform (Adaptive Biotechnologies).

TCR data were analyzed using immunoSEQ Analyzer version 3.0 (Adaptive Biotechnologies). Analysis with clonotypes was based on the amino acid sequence of the TCR. Productive rearrangements refer to the number of unique clonotypes that result in a functional TCR. Out-of-frame rearrangements are the number of unique clonotypes that result in a misaligned reading frame. Stop rearrangements are the number of unique clonotypes that contain a stop codon and do not produce a functional TCR. The number of unique clonotypes within each sample was determined based on the total number of unique clonotypes in one sample compared with another. For example, unique clonotypes found in the baseline sample were absent in the post-^90^Y sample. The complementarity-determining region 3 (CDR3) length of the TCRβ receptor was determined for each sample using the “CDR3 Length” algorithm in immunoSEQ, which calculates the CDR3 length for each clonotype and generates the productive frequency of each length in a given sample. Clonotypes unique to either the baseline or post-^90^Y samples along with those found in both (shared) were determined using the “Combined Rearrangement” function within immunoSEQ. The percentage of clonotypes that overlapped between the baseline and post-^90^Y samples was determined using the differential abundance tool which calculates the “TCR Overlap” between the two samples. TCR Overlap calculation (13) was performed in immunoSEQ. Expanding clonotypes were clonotypes whose abundance increased by >1.25-fold in the post-^90^Y sample compared with baseline. Contracting clonotypes were clonotypes whose abundances decreased >1.25-fold in the post-^90^Y sample compared with baseline. Clonotypes whose abundance did not differ between the baseline and post-^90^Y samples were termed unchanged. Clonality diversity was measured using productive Simpson clonality within immunoSEQ, which measures the richness and evenness of the T-cell repertoire in each sample.

### Statistical analysis

Data analysis was performed using JMP Pro version 17 (SAS Institute Inc.). All graphical output was generated using GraphPad Prism version 10 (GraphPad Software Inc.). Continuous variables were listed as median with IQR and categorical variables as a percentage of the total cohort. Matched pair analysis was used to determine changes in continuous variables prior to (baseline) and following ^90^Y. Bivariate analysis was performed using linear regression. Two-way ANOVA with the Sidak multiple comparisons test was used to determine significance of CDR3 length. Kaplan–Meier survival curves for TTP were generated in GraphPad Prism and compared using log-rank tests.

### Data availability

The data generated in this study are available upon request from the corresponding author.

## Results

### Study cohort

The cohort consisted of 93 patients with treatment-naïve HCC undergoing first-cycle ^90^Y. Demographics are displayed in Supplementary Table S1. In brief, 63% (59/94) of the cohort were Caucasian with a cirrhosis etiology of hepatitis C virus (HCV; 54%, 51/94), Child–Pugh A (71%, 67/94), and an Eastern Cooperative Oncology Group score of 0 (74%, 66/94). Most patients had solitary HCC burden (73%, 69/94) and were classified as BCLC stage A (82%, 77/94). All analyzed ^90^Y procedures were technically successful, with treatment characteristics and response rates displayed in Supplementary Table S2. On-protocol follow-up imaging was available for 96% (90/94) of the cohort. The target first-cycle ORR was 84% (76/90) with an oORR of 70%.

### ^90^Y-TARE induced shifts in T-cell populations

Complete blood counts were monitored prior to first-cycle ^90^Y and again at routine imaging follow-up. Treatment resulted in a posttreatment drop in both white blood cell as well as absolute lymphocyte count, whereas granulocyte, monocyte, and platelet counts remained stable (Supplementary Table S3). Although lymphopenia is an anticipated adverse event of ^90^Y, the global decrease in white blood cell seems attributable to the post-^90^Y decrease in absolute lymphocyte count.

**Figure 1:**
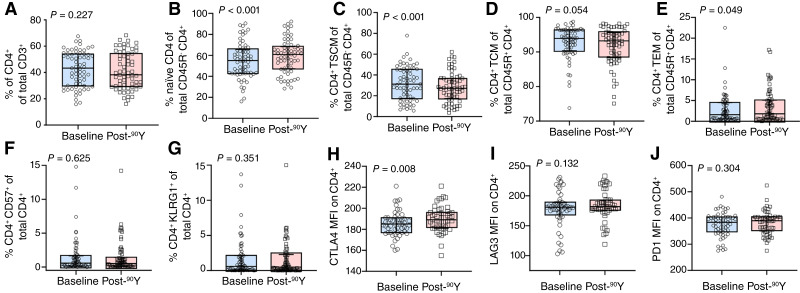
^90^Y-TARE induced changes in CD4^+^ T-cell populations in early–intermediate stage HCC. **A,** Percentage of CD4^+^ T cells of total T cells remained stable post-^90^Y, whereas **(B**) the percentage of naïve CD4^+^ increased following ^90^Y-TARE. Decreased percentage of **(C)** memory stem CD4^+^ T cells and (**D)** central memory CD4^+^ T cells was observed following ^90^Y-TARE. **E,** Increase in the percentage of effector memory/effector CD4^+^ T cells, whereas senescent T cells with (**F)** CD57^+^ and (**G)** KLRG1^+^ remained unchanged post-^90^Y. An increase in the median fluorescence intensity for the exhaustion marker (**H)** CTLA4 was observed, whereas (**I)** LAG3 and (**J)** PD1 remained stable after ^90^Y. Blue and red boxes represent IQR, and the solid horizontal line represents median. MFI, median fluorescence intensity; KLGR1, killer lectin–like receptor G1.

Immunophenotyping was performed to examine the impact of ^90^Y-induced lymphopenia within specific T-cell subsets. Supplementary Fig. S1 displays immunophenotyping panels (Supplementary Fig. S1A) designed to target naïve (Supplementary Fig. S1B and S1C), memory (Supplementary Fig. S1B and S1C), senescent (Supplementary Fig. S1D), and exhausted T-cell phenotypes (Supplementary Fig. S1E and S1F), assessed at baseline and following ^90^Y. T-cell populations were first separated based on lineage using CD4^+^ and CD8^+^ expression. Figure 1 displays shifts in CD4^+^ T-cell phenotypes at baseline and after ^90^Y. Although the percentage of CD4^+^ T cells did not change after ^90^Y (Fig. 1A), treatment induced a decrease in stem-cell memory T [TSCM; 34% (IQR, 19%–46%) vs. 27% (IQR, 18%–37%)] marked by subsequent increases in naïve [51% (IQR, 42%–63%) vs. 61% (IQR, 48%–68%)] and effector memory T [TEM; 1.5% (IQR, 0.6%–4.3%) vs. 2.1% (IQR, 0.6%–5.2%); Fig. 1B and C] cells. The percentage of central memory T (TCM) and TEM cells did not change following treatment (Fig. 1D and E). Senescent and exhaustion markers were also investigated for treatment-induced expression changes. The percentage of CD57^+^ and KLRG1^+^ CD4^+^ T cells did not change following treatment (Fig. 1F and G). Elevations of cytotoxic T-lymphocyte–associated protein 4 [CTLA4; 185 (IQR, 178–192) vs. 190 (IQR, 181–197)] were observed in CD4^+^ T cells (Fig. 1H), whereas expression of lymphocyte activation gene 3 (LAG3) and PD1 remained stable following ^90^Y (Fig 1I and J).

**Figure 2:**
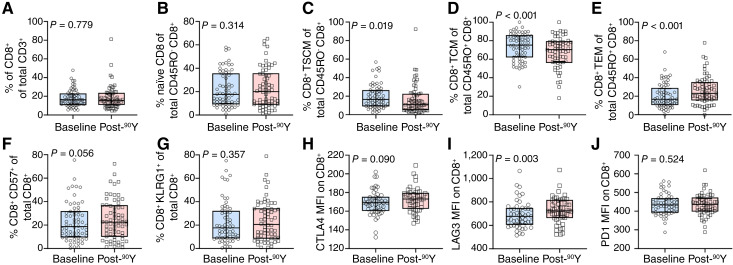
^90^Y-TARE induced changes in CD8^+^ T-cell populations in early–intermediate stage HCC. **A,** Percentage of total CD8^+^ and (**B)** naïve CD8^+^ remained stable post-^90^Y. The decreased percentage of (**C**) memory stem CD8^+^ T cells and (**D)** central memory CD8^+^ T cells was observed following ^90^Y-TARE. An increase in the percentage of (**E)** effector memory/effector CD8^+^ T cells posttreatment, whereas populations of senescent T cells that were (**F)** CD57^+^ and (**G)** KLRG1^+^ remained unchanged post-^90^Y. The median fluorescence intensity for the exhaustion marker (**H**) CTLA4 remained unchanged, whereas (**I)** LAG3 increased following treatment. MFI of (**J)** PD1 remained stable after ^90^Y. Blue and red boxes represent IQR, and the solid horizontal line represents median. MFI, median fluorescence intensity; KLGR1, killer lectin–like receptor G1.

Figure 2 displays shifts in CD8^+^ T-cell phenotypes at baseline and after ^90^Y. As observed with CD4^+^ T cells, there was no change in the percentage of CD8^+^ T cells (Fig. 2A) or naïve (Fig. 2B) after treatment. Both TSCM and TCM CD8^+^ T cells decreased after ^90^Y [Fig. 2C, 15% (IQR, 10%–25%) vs. 11% (IQR, 5%–22%) and 2D, 75% (IQR, 62%–85%) vs. 71% (IQR, 58%–79%)] with a corresponding increase in TEM cells [Fig. 2E, 18% (IQR, 11%–31%) vs. 23% (IQR, 16%–34%)] and expression of the exhaustion marker LAG3 in CD8^+^ T cells [Fig. 2I, 690 (IQR, 621–762) vs. 723 (IQR, 669–811)]. No changes were observed in the percentage of CD57^+^ and KLRG1^+^ CD8^+^ T cells (Figs. 1G and 2F) following treatment. Elevations of CTLA4 (Fig. 2H) and PD1 (Fig. 2J) expression also did not change following ^90^Y.

Upon antigen simulation, the number of effector T cells is directly correlated with a population size of TCM and TSCM found in the periphery. Active hepatitis viral infections could impact the peripheral memory T-cell populations. More than half of the study cohort had a cirrhosis etiology of HCV with a mixture of viremic (52%, 34/65) and nonviremic (48%, 31/65) infections. No correlation was found between the percentage of TSCM, TCM, or TEM cells and cirrhosis etiology nor viremic status in those with HCV infections (Supplementary Table S4). Our results suggest that after treatment with ^90^Y, there is a shift from TCM to TEM cells following ^90^Y for both CD4^+^ and CD8^+^ T cells independent of cirrhosis etiology.

### T-cell subsets and ^90^Y-TARE response rate

To answer whether certain T-cell phenotype shifts are found in ^90^Y responders versus nonresponders, patients were grouped based on target or overall response rate to first-cycle ^90^Y (Table 1; Supplementary Table S5). Patients with target NOR (target nonresponders) had a higher percentage of CD4^+^ T cells along with elevated PD1 expression on CD8^+^ T cells both before and after treatment. However, the ^90^Y-induced shifts in naïve, TSCM, TCM, and TEM cells were independent of response to treatment. Similarly, patients with overall NOR (overall nonresponders) to first-cycle ^90^Y had elevated PD1 expression on CD8^+^ T cells before and after treatment as well as a significant increase in CTLA4 expression on CD8^+^ T cells post-^90^Y (Supplementary Table S5). Taken together, although ^90^Y induces T-cell population dynamics independent of treatment response, increased and sustained expression of the T-cell exhaustion marker PD1 was the only variable prognostic for or associated with treatment response.

**Table 1:** ^90^Y induced changes in T cell phenotypes based on target response rate

	OR		NOR			Matched Pairs
T cell phenotypes	Baseline	Post-^90^Y	Baseline	Post-^90^Y	Mean difference	Mean mean
ALC, median (IQR)	1.6 (1.1–2.0)	0.8 (0.5–1.2)	1.6 (0.9–2.3)	0.7 (0.4–0.9)	0.398	0.889
CD4^+^ subpopulations						
CD4^+^ % of total CD3^+^, median (IQR)	40 (31–51)	37 (28–54)	53 (48–59)	50 (35–58)	0.134	0.019
Memory panel						
Naïve CD4^+^ % of total CD4^+^, median (IQR)	50 (42–63)	61 (47–69)	54 (43–67)	60 (55–69)	0.647	0.673
T_SCM_ CD4^+^ % of total CD4^+^, median (IQR)	35 (19–46)	27 (17–36)	32 (20–46)	27 (20–37)	0.314	0.996
T_CM_ CD4^+^ % of total CD4^+^, median (IQR)	94 (90–96)	93 (88–95)	96 (94–97)	95 (90–97)	0.408	0.340
T_EM_ CD4^+^ % of total CD4^+^, median (IQR)	1.7 (0.7–4.8)	2.3 (0.6–5.3)	0.7 (0.4–2.5)	1.0 (0.5–4.1)	0.620	0.156
Senescent panel						
CD57^+^ CD4^+^ % of total CD4^+^, median (IQR)	0.6 (0.2–2.0)	0.6 (0.09–2.2)	0.3 (0.07–1.0)	0.5 (0.02–1.0)	0.479	0.231
KLRG1^+^ CD4^+^ % of total CD4^+^, median (IQR)	0.6 (0.2–2.5)	0.6 (0.09–2.5)	0.2 (0.05–1.4)	0.4 (0.005–2.3)	0.588	0.202
Exhaustion panel						
CTLA4 on total CD4^+^, MFI, median (IQR)	189 (179–193)	188 (181–199)	180 (175–187)	191 (185–196)	0.159	0.197
LAG3 on total CD4^+^, MFI, median (IQR)	182 (175–191)	182 (177–197)	177 (165–189)	181 (165–196)	0.545	0.458
PD-1 on total CD4^+^, MFI, median (IQR)	382 (343–406)	386 (344–404)	384 (356–394)	399 (364–423)	0.797	0.374
CD8^+^ subpopulations						
CD8^+^ % of total CD3^+^, median (IQR)	16 (12–23)	16 (11–23)	15 (11–21)	15 (10–19)	0.403	0.176
Memory panel						
Naïve CD8^+^ % of total CD8^+^, median (IQR)	15 (9–32)	16 (9–35)	21 (16–36)	23 (12–34)	0.687	0.809
T_SCM_ CD8^+^ % of total CD8^+^, median (IQR)	15 (10–24)	11 (5–21)	21 (13–44)	19 (9–34)	0.796	0.143
T_CM_ CD8^+^ % of total CD8^+^, median (IQR)	73 (61–84)	70 (57–79)	83 (62–89)	78 (65–81)	0.360	0.632
T_EM_ CD8^+^ % of total CD8, median (IQR)	18 (12–31)	24 (16–35)	12 (8–30)	20 (15–32)	0.564	0.721
Senescent panel						
CD57^+^ CD8^+^ % of total CD8^+^, median (IQR)	21 (12–34)	22 (11–37)	12 (5–24)	25 (4–38)	0.353	0.207
KLRG1^+^ CD8^+^ % of total CD8^+^, median (IQR)	20 (11–34)	20 (9–37)	13 (8–24)	20 (5.8–34)	0.281	0.212
Exhaustion panel						
CTLA4 on total CD8^+^, MFI, median (IQR)	171 (164–177)	174 (163–180)	162 (158–169)	170 (163–182)	0.089	0.646
LAG3 on total CD8^+^, MFI, median (IQR)	696 (612–762)	726 (669–817)	668 (613–765)	710 (664–794)	0.982	0.647
PD-1 on total CD8^+^, MFI, median (IQR)	434 (400–472)	420 (395–450)	464 (436–511)	483 (440–521)	0.397	0.003

Abbreviations: ALC, absolute lymphocyte count; CTLA4, cytotoxic T-lymphocyte associated protein 4; IQR, interquartile range; KLGR1, killer lectin-like receptor G1; LAG3, lymphocyte activation gene 3; MFI, median fluorescence intensity; NOR, non-objective response rate; ORR, objective response rate; PD-1, programmed cell death-1; T_CM_, central memory T cell; T_EM_, effector memory T cells; T_SCM_, memory stem T cell; ^90^Y, Yttrium-90.

### Longitudinal TCR rearrangement and clonotype profiles following ^90^Y

Although immunophenotyping data suggest a ^90^Y-induced effector shift in the T-cell population, the circulating TCR repertoire provides a means to identify and characterize potential neoantigen responses as well as assess treatment response–stratified changes in T-cell populations. The TCRs of peripheral T cells were sequenced in 75 patients prior to treatment with first-cycle ^90^Y (baseline sample) and following treatment (post-^90^Y sample) and were then analyzed by clonotype to group T cells with the same amino acid sequence in the TCR. Two patient samples (one baseline and one post-^90^Y sample) had incomplete TCR sequencing data due to insufficient T cells available for sequencing. The population characteristics for TCR rearrangements at treatment baseline and post-^90^Y are displayed in Supplementary Table S6. The median number of T cells sequenced for baseline samples was 53,600 (IQR, 27,000–85,700). Productive rearrangements resulting in a functional TCR were present in 82% of the T cells with the remaining T cells containing nonfunctional TCRs created by out-of-frame rearrangements (17%) and stop rearrangements (1.4%). Of the T cells with productive rearrangements, 70% had a unique clonotype (TCR amino acid sequence) representing 57% of the total T-cell population.

The derivation of T cells in the sample from the TCR sequencing results confirmed the ^90^Y-induced decrease in circulating T cells at imaging follow-up (Supplementary Table S6). Although paired analysis revealed significantly lower total productive, out-of-frame, and stop rearrangements along with unique clonotypes after ^90^Y, the fractional percentage remained stable (Supplementary Table S6). This suggests that the treatment-induced changes in the T-cell compartment did not impact the overall rearrangements observed within the repertoire or the fraction of unique clones within the population.

CDR3 length was investigated at baseline and following ^90^Y to determine whether there was a treatment effect. Both CDR3 lengths at baseline and post-^90^Y for each patient were determined using the sum frequency of clonotypes for each CDR3 length (30–60 amino acids). CDR3 length for clonotypes did not change post-^90^Y (Supplementary Fig. S2).

### Clonotypes within HCC prior to and following ^90^Y

The top 10 clonotypes present in baseline samples are shown in Supplementary Table S7. Overall, a single clonotype (CASSLGETQYF) was found in 85% (62/73) of samples at baseline prior to any treatment. Three clonotypes were found in 78% (57/73) of baseline samples. The predominant clonotype by cirrhosis etiology and HCV viral status was analyzed to isolate potential unique sequence signatures. The top five clonotypes based on cirrhosis etiology are displayed in Supplementary Table S8. Briefly, patients with HCV shared one clonotype (CASSLGETQYF) in 85% (45/53) of samples, although this sequence was also the most common in the overall cohort. Two clonotypes were present in 81% (43/53) of patients with HCV at baseline. Although 17 patients had SLD, one clonotype (CASSPSTDTQYF) was present in 82% (14/17) and four clonotypes were shared in 71% (12/17) of patients (Supplementary Table S8).

The top 10 clonotypes post-^90^Y are displayed in Supplementary Table S9. The clonotype (CASSLGETQYF) shared in most baseline samples was also the most shared clonotype following ^90^Y and found in 88% (64/73) of patients. This same clonotype was shared in 94% (16/17) of patients with SLD and in 87% (46/53) of patients with HCV (Supplementary Table S8).

### Effect of ^90^Y on TCR repertoire

**Figure 3:**
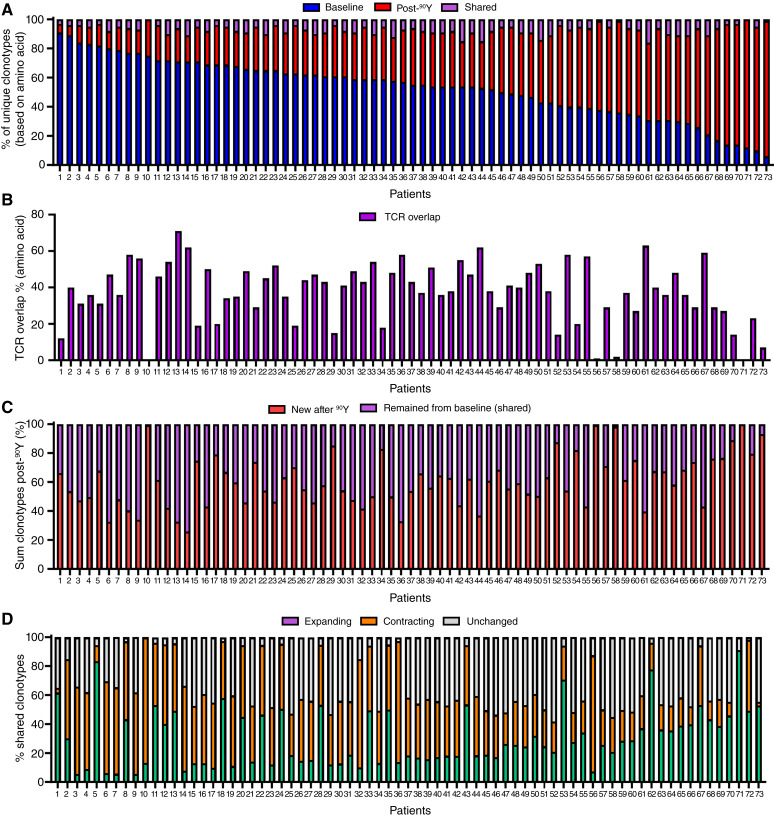
^90^Y-TARE induces new clonotypes and the expansion and contraction of existing clonotypes. **A,** Unique clonotypes in the baseline (blue) and post-^90^Y (red) and shared clonotypes between baseline and post-^90^Y (purple). **B,** Percentage of the TCR repertoire that overlaps between baseline and post-^90^Y. **C,** Sum frequency of clonotypes post-^90^Y that were new (red) or remained from baseline (purple). **D,** Percentage of clonotypes shared between baseline and post-^90^Y that were either expanding (green), contracting (orange), or remained unchanged (gray).

Although productive rearrangement and unique clonotype frequencies were similar following ^90^Y, the posttreatment expansion in effector memory populations suggests that longitudinal changes within the clonotype fractions may be present despite the similar overall frequency of unique clonotypes. Specifically, these longitudinal changes may include unique clonotypes that are absent in the baseline sample and induced following ^90^Y. Using the baseline samples as reference, the number of unique clonotypes as well as those shared between the baseline and post-^90^Y samples was calculated for each patient (Fig. 3A). The generation of new clonotypes following treatment was highly variable across the cohort. Unique TCR clonotypes found only in the baseline sample accounted for 55% (IQR, 39%–69%) of total TCR clonotypes. However, the median percentage of unique clonotypes that arose post-^90^Y was 36% (IQR, 25%–55%). Forty-six patients (46/73) had ≥50% of unique clonotypes within the baseline sample. In the post-^90^Y sample, 22 patients (22/73) had ≥50% of unique clonotypes, indicating a large amount of new clonotypes in circulation following treatment. Interestingly, only 6% (IQR, 5%–9%) of unique clonotypes persistent from baseline and following treatment, indicating vast changes in the peripheral TCR repertoire post-^90^Y.

Figure 3A characterizes the clonotype distribution based on their detection across both samples, but it does not account for the clonotype frequency in those samples. Although the number of unique clonotypes shared between samples was small (6%), those shared sequences made up 38% (IQR, 29%–49%) of the sample overlapping repertoire (Fig. 3B). These shared clonotype frequencies were then compared with the fractional share of the new clonotypes generated after ^90^Y in the follow-up samples (Fig. 3C). The shared clonotypes made up 40% (IQR, 28%–52%) of the post-^90^Y TCR repertoire with the new clonotypes representing the majority component of the repertoire. Potential neoantigens could also be present in the shared TCR repertoire and could be reactivated following treatment. Figure 3D characterizes the fractional percentage of shared TCR clonotypes categorized as expanding (>1.25-fold increase), contracting (<1.25-fold decrease), or stable (fold change <±1.25). Stable (median, 42%; IQR, 6%–47%) and contracting (median, 39%; IQR, 23%–47%) clonotypes made up the major fractions with expanding clonotypes having the smallest fraction (median, 26%; IQR, 14%–45%), but with significant patient-to-patient variation. Collectively, the longitudinal clonotype analysis supports a neoantigen response that is highly variable and could contain clonotypes reactivated during treatment.

Clonotype dynamics were then examined for concordance with tORR and oORR (Table 2). The percentages of unique clonotypes within each sample (baseline, post-^90^Y, or shared) were not associated with tORR or oORR.

**Table 2:** Unique T cell receptor clonotypes based on ^90^Y response rate

	Target ORR			Overall ORR		
Unique TCR clonotypes (% of total)	OR	NOR	*P* value	OR	NOR	*P* value
Baseline, median (IQR)	55 (37–67)	55 (41–69)	0.401	54 (37–70)	59 (41–68)	0.352
Post-^90^Y, median (IQR)	36 (27–57)	32 (23–55)	0.371	37 (25–56)	32 (25–55)	0.382
Shared, median (IQR)	7 (5–9)	6 (5–9)	0.730	7 (4–9)	6 (5–9)	0.779

Abbreviations: IQR, interquartile range; NOR, non-objective response; OR, objective response; ORR, objective response rate; TCR, T cell receptor; ^90^Y, Yttrium-90.

### TCR repertoire clonality and T-cell phenotypes

**Figure 4:**
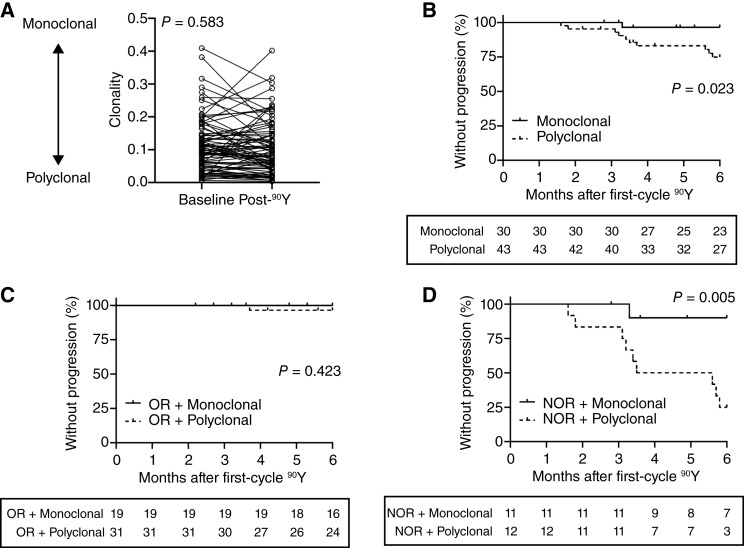
TCR repertoire clonality and impacts on TTP. **A,** Changes in TCR repertoire measured as clonality from baseline to post-^90^Y. Shifts in the T-cell repertoire toward more monoclonal or polyclonal were determined by comparing clonality changes from baseline to post-^90^Y. **B,** Patients with shifts toward more polyclonal TCR repertoires had shorter rapid TTP. **C,** Patients with ORs to ^90^Y had excellent TTP. **D,** Patients with NORs to ^90^Y and shifts toward a polyclonal TCR repertoire had worse TTP.

Monitoring clonotype frequencies provides evidence of changes within the peripheral TCR repertoire while identifying potential sequences associated with durable antitumoral responses. The TCR repertoire clonality calculation following treatment can provide an overall determination of unique clonotype abundance and characterize the circulating T-cell pool as polyclonal verses monoclonal. Overall, ^90^Y did not induce a change in population clonality (Fig. 4A). However, a subgroup of patients (36%, 26/73) showed marked increases in polyclonal or monoclonal responses characterized by a shift of clonality >5%.

### Post-^90^Y TCR repertoire and TTP

Both the longitudinal immunophenotyping and TCR repertoire analysis support that ^90^Y activates existing and stimulates new clonotypes following treatment but in a manner not directly associated with the initial target or overall response to treatment. An objective ^90^Y response, specifically first-cycle OR and durable overall OR, are strongly prognostic of HCC outcomes. Peripheral clonality and tumoral clonality are the most common data outputs from TCR sequencing analysis in studies of human cancer. Peripheral clonality was analyzed for the total cohort and then stratified based on oORR to compare outcomes associated with a posttreatment monoclonal or polyclonal TCR repertoire (Fig. 4B and D). A polyclonal profile stratified patients with a higher risk of disease progression to BCLC-C (Fig. 4B) within 6 months post-^90^Y (*P* = 0.023; HR, 7.5). In patients with a first-cycle response to ^90^Y (Fig. 4C), 6-month outcomes were excellent and not stratified by peripheral clonality (*P* = 0.423). However, nonresponders with a polyclonal profile were at increased risk of disease progression (*P* = 0.005; HR, 10.8) with nearly 75% experiencing disease progression within 6 months after treatment (Fig. 4D).

## Discussion

^90^Y is an emerging strategy for definitive treatment and bridge/downstage to surgery in HCC with BCLC stages A–B and Eastern Cooperative Oncology Group score of 0 to 1 (14). Excellent target response rates (4) and potential to improve response rates in advanced stage HCC in combination with ICI have been reported in which the treatment responder population has been difficult to predict. There is a great need to understand the immunologic impact of ^90^Y to understand how this treatment affects antitumoral T-cell populations and potentially uncover which patients would benefit from the addition of ICI. ^90^Y treatment is characterized by a transient period of posttreatment lymphopenia (15) that is associated with the lung shunt fraction and not with response to treatment or HCC-specific outcomes (8). However, T-cell lineages or phenotypes that are most affected by ^90^Y remain unclear. In this study, T-cell subsets and TCR repertoire were examined before and after ^90^Y in which treatment demonstrated distinct evidence of the immunologic response in peripheral T cells.

^90^Y has been shown to activate T cells both within the tumor microenvironment (TME) and in the periphery (9, 10), providing evidence of immunomodulation. However, TME evidence is limited to postresected BCLC-0 (very early) tumors evaluated more than 6 months after ^90^Y and in the absence of cirrhosis-associated immune dysfunction. Posttreatment tumor sampling is contraindicated in BCLC A–B due to the risk of tumor-related complications including track seeding. Therefore, immune monitoring strategies must ideally use longitudinal, noninvasive sampling to characterize the T-cell responses. Prior evidence confirms that peripheral T cells provide insights to TME response and serve as biomarkers for ICI response (16–18). Overlap of TCR repertoire and tumor antigen specificities was found between the periphery and the TME in patients with melanoma (19), providing further evidence of the correlation between the periphery and TME immune landscape.

Memory T-cell subsets (stem, central, and effector memory) are central to the antitumoral effector response and serve as a critical biomarker for ICI response (see review ref. 20). The first cycle of ^90^Y induced a CD8^+^ TEM response in 66% patients at a median follow-up of 75 days. TEM cells recirculate in the blood, traffic to peripheral tissues (21), and produce antitumoral effector molecules upon antigen-specific activation (22). Although TEM cells are more durable and rapidly secrete larger amounts of IFNγ and perforin upon activation compared with TCM cells (23, 24), in mouse models, TCM cells have been shown to have better antitumoral activity (25). TCM cells are denoted for expressing lymph node homing properties through CCR7 expression compared with TEM cells (23), which are CCR7^−^. Whether the decrease in TCM cells post-^90^Y was due to differentiation into TEM cells or whether activation led to relocation to the lymph nodes is unknown. Regardless, the effects of ^90^Y on circulating memory T cells were independent of cirrhosis etiology, the degree of lymphopenia, and treatment response.

Rivoltini and colleagues (10) similarly reported an increase in the proliferation marker Ki67 and the T-cell effector molecule granzyme B 1 month following ^90^Y, supporting a treatment-induced effector response. Their results also captured the post-^90^Y exhaustion signature of CTLA4 and LAG3 (10) observed at treatment follow-up in this study. Their study identified only T-cell population (CD4^+^Ki67^+^GranB^+^) to be associated with treatment response, with the posttreatment immune profile not ultimately found to be predictive of long-term disease control, in agreement with our results. The difference in treatment prognostic immune populations could be linked to differences in disease staging (BCLC B–C vs. BCLC A–B) and the corresponding ORR (unreported ∼65% vs. 84%).

Sustained CD8^+^ PD1 elevation remained the dominant immunologic risk factor for NOR, consistent with our prior results in early-stage HCC treated with liver-directed therapy (8, 26). Elevated PD1 expression in these patients is notably sustained beyond the overall peripheral PD1 burst effect described by Rivoltini and colleagues 1 month following ^90^Y. The sustained expression of PD1 in these patients could indicate a treatment responder population who may benefit from combination ^90^Y–ICI directly targeting T-cell exhaustion (PD1, CTLA4, LAG3, and TGIT). To make matters more challenging, HCC is known to have different immune signatures (27) likely due to underlying etiology and cirrhosis-associated immune dysfunction, which may impact which patients express an exhausted phenotype within the TME. Although overall response rates for anti-PD1 have remained low (<20%; ref. 28), perhaps selection of patients expressing PD1 would improve response rates.

Definitive treatment in ^90^Y aims to eradicate tumor burden while eliciting an immunogenic response to promote a durable antitumoral response. Although ablation has been shown to release tumor-specific antigens (TSA) in HCC (29), studies of TSA release following ^90^Y are lacking. The TCR repertoire holds key information on antigen exposure and could provide insights into TSA release with TCR sequencing serving to identify antigen biomarker signatures associated with durable treatment outcomes. Neither ^90^Y treatment nor the lymphodepleting effect of ^90^Y impaired the generation of functional TCRs or the percentage of unique clonotypes in the circulating T-cell population. The TCR repertoire was more diverse at treatment baseline with roughly 1/3 of the post-^90^Y repertoire representing unique clonotypes with respect to baseline, although the degree of response widely varied across the cohort. The generation of new clonotypes post-^90^Y was not impacted by treatment-induced lymphopenia. Surprisingly, only 6% of the TCR repertoire diversity persisted from baseline to post-^90^Y. However, these persistent clonotypes represented 40% of the post-^90^Y TCR repertoire although they also widely varied from patient to patient.

Although a dynamic T-cell response was confirmed by TCR sequencing, the frequency and abundance of new and expanding clonotypes were highly variable and ultimately not associated with ORR. Currently, there are no strategies to identify potential tumor antigens based on the clonotype sequences, though new technology aimed at antigen recognition from TCR sequences is under development. Recent studies have paired peripheral blood and tumor site TCR analysis to identify potential neoantigen or reactivated tumor antigen sequences (30, 31). However, a benefit of the high ORR to ^90^Y is that potential treatment-enriched candidate sequences were identified, notably one sequence (CASSLGETQYF) was present in 88% of post-^90^Y samples. These sequences could provide additional targets for chimeric antigen receptor T-cell therapy for HCC, whereas current clinical trials have focused on the protein glypican 3 and have not been completed (32).

TCR clonality offers a way to evaluate the diversity of the TCR repertoire and monitor T-cell dynamics upon interaction with an antigen. Clonality is the most commonly prognostic variable in sequencing analysis, particularly those focused on ICI response in various cancers. TCR clonality in the periphery and tumor showed a strong overlap in melanoma (33–35) as well as in HCC (30, 31). Overall, the prognostic implications of clonal expansion versus population diversity have been inconsistent in the literature (11) and likely depend specifically upon the TSA pool within the repertoire and population sequenced. ^90^Y did not induce an overall change in repertoire clonality, although a subgroup of patients had notable shifts in clonality or diversity independent of treatment response rate. When the response rate was used as a subgrouping variable, polyclonal expansion emerged as a critical risk for impending rapid disease progression to BCLC-C. In patients with an OR and very low risk of rapid disease progression, posttreatment clonality and diversity were irrelevant and may play a major role over the course of extended follow-up. The monoclonal TCR response may indicate immune activation in a subset of TSA-specific T cells. Patients with melanoma with higher tumoral clonality and more monoclonal intratumoral TCR repertories had higher responses rates to anti-PD1 therapy (36, 37). In HCC, one study found more monoclonal intratumoral TCR clonality and T-cell fractions in patients responding to anti-CTLA4 and ablation (17). The result from this study suggests that a polyclonal repertoire coupled with a lack of treatment response portend a high disease progression risk and may identify a potential treatment responder group to combine with ICI.

There are some limitations in this study. ^90^Y follow-up was collected at the time of imaging follow-up and varied among patients based upon the need to expedite sequential therapy or other unforeseeable circumstances. Memory T-cell phenotypes were, therefore, prioritized over TEM cell analysis, but it is recognized that these populations may remain in circulation beyond the prime response window identified by Rivoltini and colleagues. Although TCR sequencing was strengthened by pairing a treatment baseline, sequencing was performed on the isolated peripheral T cells without sorting based on the memory T-cell phenotype. Isolating the TCR repertoire of the TEM cell fraction would increase the relevance of the isolated sequences with an equal importance of evaluating the sequences associated with sustained elevation in PD1 linked to the treatment nonresponse rate.

In conclusion, T-cell lineage shifts were observed following first-cycle ^90^Y with TCR sequencing analysis confirming dynamic immune fluctuations following treatment. A larger database of peripheral T cells and tumor-infiltrating lymphocyte sequences associated with response rate and durable HCC treatment outcomes may help identify targetable TSA to globally improve response rates. The polyclonal immune signature in patients not responding to first-cycle ^90^Y and associated rapid disease progression as well as inferior outcomes associated with sustained PD1 expression suggests a target population in BCLC A–B who may benefit from an immediate switch to ICI.

## Supplementary Material

Supplementary Figure 1 Supplemental Figure 1

Supplementary Figure 2 Supplemental Figure 2

Supplemental tables Supplemental tables 1 to 9

## References

[1] Villanueva A . Hepatocellular carcinoma. N Engl J Med 2019;380:1450–62. 30970190 10.1056/NEJMra1713263

[2] Akinyemiju T , Abera S, Ahmed M, Alam N, Alemayohu MA, Allen C, ; Global Burden of Disease Liver Cancer Collaboration. The burden of primary liver cancer and underlying etiologies from 1990 to 2015 at the global, regional, and national level: results from the global burden of disease study 2015. JAMA Oncol 2017;3:1683–91. 28983565 10.1001/jamaoncol.2017.3055 PMC5824275

[3] Younossi Z , Stepanova M, Ong JP, Jacobson IM, Bugianesi E, Duseja A, . Nonalcoholic steatohepatitis is the fastest growing cause of hepatocellular carcinoma in liver transplant candidates. Clin Gastroenterol Hepatol 2019;17:748–55.e3. 29908364 10.1016/j.cgh.2018.05.057

[4] Salem R , Johnson GE, Kim E, Riaz A, Bishay V, Boucher E, . Yttrium-90 radioembolization for the treatment of solitary, unresectable HCC: the LEGACY study. Hepatology 2021;74:2342–52. 33739462 10.1002/hep.31819 PMC8596669

[5] Kim E , Sher A, Abboud G, Schwartz M, Facciuto M, Tabrizian P, . Radiation segmentectomy for curative intent of unresectable very early to early stage hepatocellular carcinoma (RASER): a single-centre, single-arm study. Lancet Gastroenterol Hepatol 2022;7:843–50. 35617978 10.1016/S2468-1253(22)00091-7

[6] Finn RS , Qin S, Ikeda M, Galle PR, Ducreux M, Kim TY, . Atezolizumab plus bevacizumab in unresectable hepatocellular carcinoma. N Engl J Med 2020;382:1894–905. 32402160 10.1056/NEJMoa1915745

[7] Kudo M . Durvalumab plus tremelimumab in unresectable hepatocellular carcinoma. Hepatobiliary Surg Nutr 2022;11:592–6. 36016731 10.21037/hbsn-22-143 PMC9396100

[8] Núñez KG , Sandow T, Gimenez J, Hibino M, Fort D, Cohen AJ, . Lineage-specific regulation of PD-1 expression in early-stage hepatocellular carcinoma following 90yttrium transarterial radioembolization - implications in treatment outcomes. Eur J Cancer 2024;196:113442. 37988841 10.1016/j.ejca.2023.113442

[9] Chew V , Lee YH, Pan L, Nasir NJM, Lim CJ, Chua C, . Immune activation underlies a sustained clinical response to Yttrium-90 radioembolisation in hepatocellular carcinoma. Gut 2019;68:335–46. 29440463 10.1136/gutjnl-2017-315485 PMC6352403

[10] Rivoltini L , Bhoori S, Camisaschi C, Bergamaschi L, Lalli L, Frati P, . Y^90^-radioembolisation in hepatocellular carcinoma induces immune responses calling for early treatment with multiple checkpoint blockers. Gut 2023;72:406–7. 35508369 10.1136/gutjnl-2021-326869 PMC9872224

[11] Kidman J , Principe N, Watson M, Lassmann T, Holt RA, Nowak AK, . Characteristics of TCR repertoire associated with successful immune checkpoint therapy responses. Front Immunol 2020;11:587014. 33163002 10.3389/fimmu.2020.587014 PMC7591700

[12] Llovet JM , Lencioni R. mRECIST for HCC: performance and novel refinements. J Hepatol 2020;72:288–306. 31954493 10.1016/j.jhep.2019.09.026 PMC12452114

[13] Emerson RO , Sherwood AM, Rieder MJ, Guenthoer J, Williamson DW, Carlson CS, . High-throughput sequencing of T-cell receptors reveals a homogeneous repertoire of tumour-infiltrating lymphocytes in ovarian cancer. J Pathol 2013;231:433–40. 24027095 10.1002/path.4260 PMC5012191

[14] Reig M , Forner A, Rimola J, Ferrer-Fàbrega J, Burrel M, Garcia-Criado Á, . BCLC strategy for prognosis prediction and treatment recommendation: the 2022 update. J Hepatol 2022;76:681–93. 34801630 10.1016/j.jhep.2021.11.018 PMC8866082

[15] Riaz A , Awais R, Salem R. Side effects of yttrium-90 radioembolization. Front Oncol 2014;4:198. 25120955 10.3389/fonc.2014.00198 PMC4114299

[16] Manjarrez-Orduño N , Menard LC, Kansal S, Fischer P, Kakrecha B, Jiang C, . Circulating T cell subpopulations correlate with immune responses at the tumor site and clinical response to PD1 inhibition in non-small cell lung cancer. Front Immunol 2018;9:1613. 30123214 10.3389/fimmu.2018.01613 PMC6085412

[17] Agdashian D , ElGindi M, Xie C, Sandhu M, Pratt D, Kleiner DE, . The effect of anti-CTLA4 treatment on peripheral and intra-tumoral T cells in patients with hepatocellular carcinoma. Cancer Immunol Immunother 2019;68:599–608. 30688989 10.1007/s00262-019-02299-8 PMC6662600

[18] Fairfax BP , Taylor CA, Watson RA, Nassiri I, Danielli S, Fang H, . Peripheral CD8^+^ T cell characteristics associated with durable responses to immune checkpoint blockade in patients with metastatic melanoma. Nat Med 2020;26:193–9. 32042196 10.1038/s41591-019-0734-6 PMC7611047

[19] Gros A , Parkhurst MR, Tran E, Pasetto A, Robbins PF, Ilyas S, . Prospective identification of neoantigen-specific lymphocytes in the peripheral blood of melanoma patients. Nat Med 2016;22:433–8. 26901407 10.1038/nm.4051 PMC7446107

[20] Han J , Khatwani N, Searles TG, Turk MJ, Angeles CV. Memory CD8^+^ T cell responses to cancer. Semin Immunol 2020;49:101435. 33272898 10.1016/j.smim.2020.101435 PMC7738415

[21] Farber DL , Yudanin NA, Restifo NP. Human memory T cells: generation, compartmentalization and homeostasis. Nat Rev Immunol 2014;14:24–35. 24336101 10.1038/nri3567 PMC4032067

[22] Principe N , Kidman J, Goh S, Tilsed CM, Fisher SA, Fear VS, . Tumor infiltrating effector memory antigen-specific CD8^+^ T cells predict response to immune checkpoint therapy. Front Immunol 2020;11:584423. 33262762 10.3389/fimmu.2020.584423 PMC7688517

[23] Sallusto F , Lenig D, Förster R, Lipp M, Lanzavecchia A. Two subsets of memory T lymphocytes with distinct homing potentials and effector functions. Nature 1999;401:708–12. 10537110 10.1038/44385

[24] Tussey L , Speller S, Gallimore A, Vessey R. Functionally distinct CD8^+^ memory T cell subsets in persistent EBV infection are differentiated by migratory receptor expression. Eur J Immunol 2000;30:1823–9. 10940871 10.1002/1521-4141(200007)30:7<1823::AID-IMMU1823>3.0.CO;2-6

[25] Hinrichs CS , Spolski R, Paulos CM, Gattinoni L, Kerstann KW, Palmer DC, . IL-2 and IL-21 confer opposing differentiation programs to CD8^+^ T cells for adoptive immunotherapy. Blood 2008;111:5326–33. 18276844 10.1182/blood-2007-09-113050 PMC2396726

[26] Núñez KG , Sandow T, Fort D, Hibino M, Wright P, Cohen AJ, . PD-1 expression in hepatocellular carcinoma predicts liver-directed therapy response and bridge-to-transplant survival. Cancer Immunol Immunother 2022;71:1453–65. 34689234 10.1007/s00262-021-03087-z PMC9122885

[27] Sia D , Jiao Y, Martinez-Quetglas I, Kuchuk O, Villacorta-Martin C, Castro de Moura M, . Identification of an immune-specific class of hepatocellular carcinoma, based on molecular features. Gastroenterology 2017;153:812–26. 28624577 10.1053/j.gastro.2017.06.007 PMC12166766

[28] El-Khoueiry AB , Sangro B, Yau T, Crocenzi TS, Kudo M, Hsu C, . Nivolumab in patients with advanced hepatocellular carcinoma (CheckMate 040): an open-label, non-comparative, phase 1/2 dose escalation and expansion trial. Lancet 2017;389:2492–502. 28434648 10.1016/S0140-6736(17)31046-2 PMC7539326

[29] Mizukoshi E , Yamashita T, Arai K, Sunagozaka H, Ueda T, Arihara F, . Enhancement of tumor-associated antigen-specific T cell responses by radiofrequency ablation of hepatocellular carcinoma. Hepatology 2013;57:1448–57. 23174905 10.1002/hep.26153

[30] Zheng C , Zheng L, Yoo JK, Guo H, Zhang Y, Guo X, . Landscape of infiltrating T cells in liver cancer revealed by single-cell sequencing. Cell 2017;169:1342–56.e16. 28622514 10.1016/j.cell.2017.05.035

[31] Sing GK , Li D, Chen X, Macnaughton T, Lichanska AM, Butterworth L, . A molecular comparison of T lymphocyte populations infiltrating the liver and circulating in the blood of patients with chronic hepatitis B: evidence for antigen-driven selection of a public complementarity-determining region 3 (CDR3) motif. Hepatology 2001;33:1288–98. 11343258 10.1053/jhep.2001.24026

[32] Ozer M , Goksu SY, Akagunduz B, George A, Sahin I. Adoptive cell therapy in hepatocellular carcinoma: a review of clinical trials. Cancers (Basel) 2023;15:1808. 36980692 10.3390/cancers15061808 PMC10046758

[33] Arakawa A , Vollmer S, Tietze J, Galinski A, Heppt MV, Bürdek M, . Clonality of CD4^+^ blood T cells predicts longer survival with CTLA4 or PD-1 checkpoint inhibition in advanced melanoma. Front Immunol 2019;10:1336. 31275310 10.3389/fimmu.2019.01336 PMC6591437

[34] Lucca LE , Axisa P-P, Lu B, Harnett B, Jessel S, Zhang L, . Circulating clonally expanded T cells reflect functions of tumor-infiltrating T cells. J Exp Med 2021;218:e20200921. 33651881 10.1084/jem.20200921 PMC7933991

[35] Fehlings M , Kim L, Guan X, Yuen K, Tafazzol A, Sanjabi S, . Single-cell analysis reveals clonally expanded tumor-associated CD57^+^ CD8 T cells are enriched in the periphery of patients with metastatic urothelial cancer responding to PD-L1 blockade. J Immunother Cancer 2022;10:e004759. 35981786 10.1136/jitc-2022-004759 PMC9394212

[36] Tumeh PC , Harview CL, Yearley JH, Shintaku IP, Taylor EJ, Robert L, . PD-1 blockade induces responses by inhibiting adaptive immune resistance. Nature 2014;515:568–71. 25428505 10.1038/nature13954 PMC4246418

[37] Valpione S , Mundra PA, Galvani E, Campana LG, Lorigan P, De Rosa F, . The T cell receptor repertoire of tumor infiltrating T cells is predictive and prognostic for cancer survival. Nat Commun 2021;12:4098. 34215730 10.1038/s41467-021-24343-x PMC8253860

