# Supplementary material for: Yttrium-90 Induces an Effector Memory Response with Neoantigen Clonotype Expansion: Implications for Immunotherapy
Source: Cancer Res Commun. 2024 Aug 19;4(8):2163–73. doi: 10.1158/2767-9764.CRC-24-0228 (PMC11331567; doi:10.1158/2767-9764.CRC-24-0228)
Supplement: Supplementary Figure 1 — Supplemental Figure 1 [file crc-24-0228_supplementary_figure_1_supps1.docx]

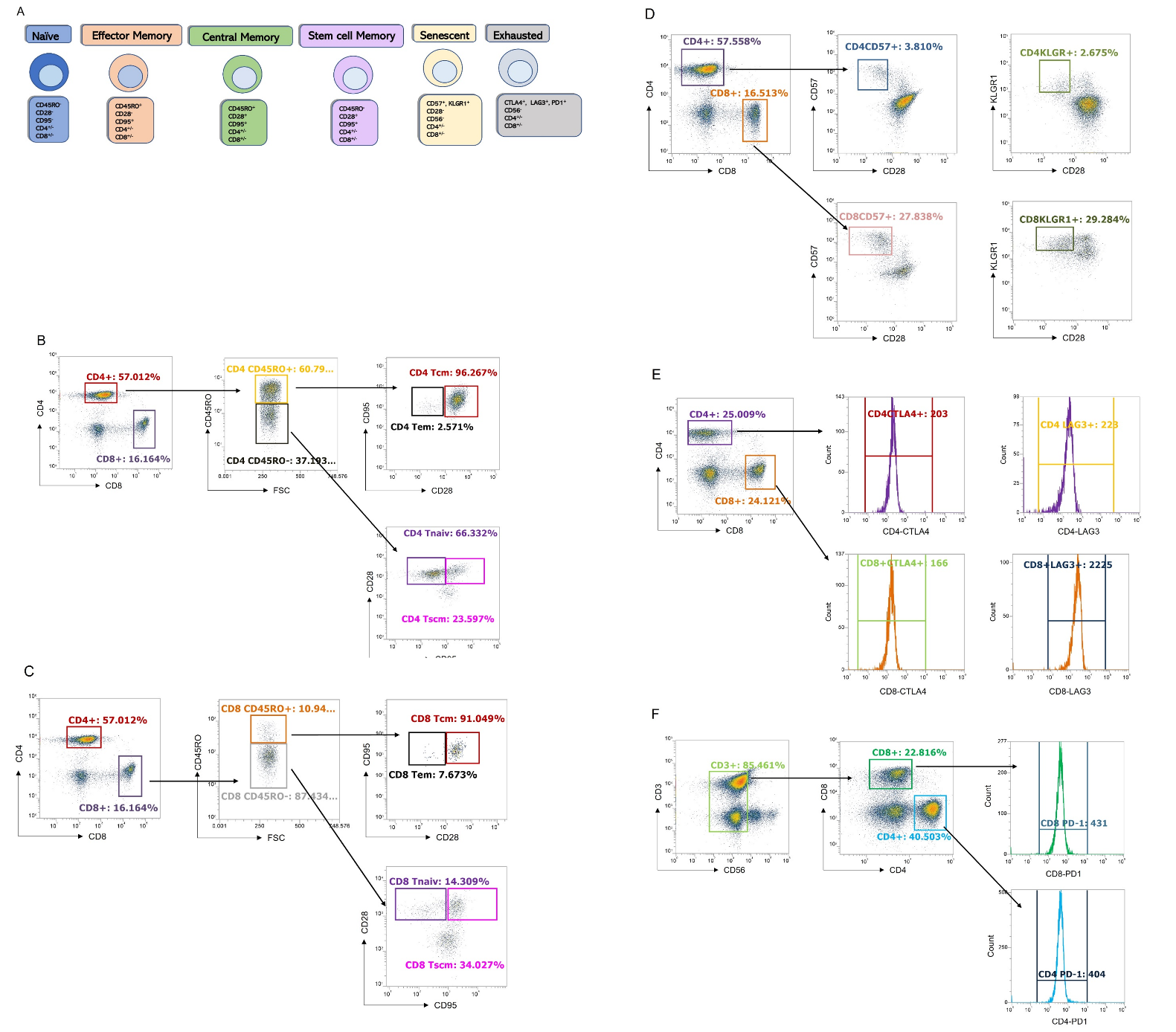


**Supplemental Figure 1**. **T Cell Population Flow Panel and Gating Strategies.** (A) Surface markers for flow cytometry panel for each T cell population. Gating strategy for memory panel that included naïve, central memory, stem cell memory, and effector memory for (B) CD4^+^ and (C) CD8^+^ T cells. (D) Gating strategy for markers of senescence, CD57 and KLGR1 on CD4^+^ and CD8^+^ T cells. (E) Gating strategy to measure median fluorescence intensity of PD-1 on CD4^+^ and CD8^+^ T cells. (F) Gating strategy for exhaustion markers CTLA4 and LAG3 measured as median fluorescence intensity on CD4^+^ and CD8^+^ T cells. Abbreviations: Naïve T cell (T_Naiv_), Central memory T cell (T_CM_), Stem cell memory (T_SCM_), Effector memory T cells (T_EM_), Programmed cell death protein-1 (PD-1), cytotoxic T-lymphocyte associated protein 4 (CTLA4), Lymphocyte activation gene 3 (LAG3), Killer cell lectin like receptor G1 (KLGR1).
