# Supplementary material for: Yttrium-90 Induces an Effector Memory Response with Neoantigen Clonotype Expansion: Implications for Immunotherapy
Source: Cancer Res Commun. 2024 Aug 19;4(8):2163–73. doi: 10.1158/2767-9764.CRC-24-0228 (PMC11331567; doi:10.1158/2767-9764.CRC-24-0228)
Supplement: Supplementary Figure 2 — Supplemental Figure 2 [file crc-24-0228_supplementary_figure_2_supps2.docx]

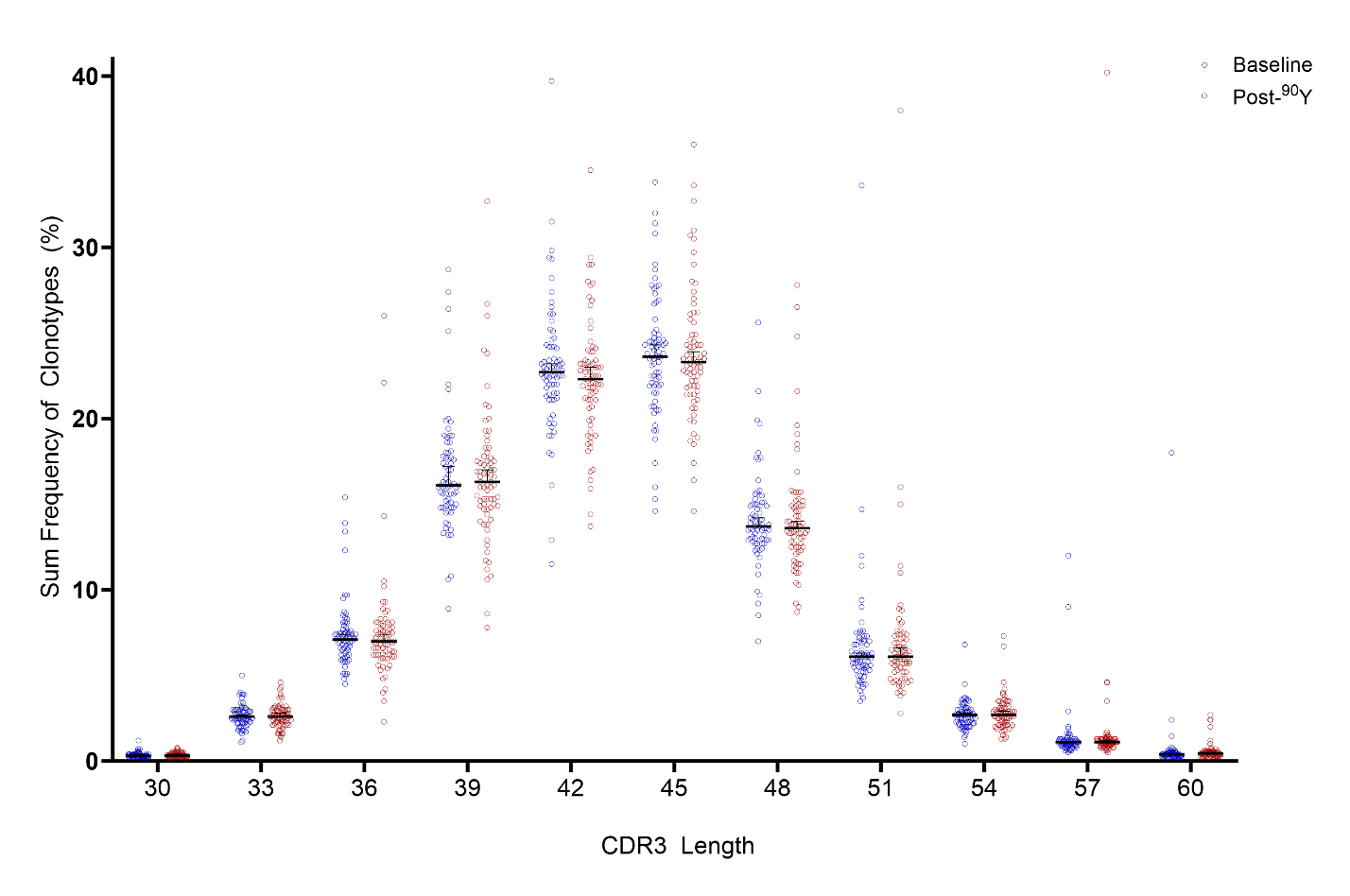


**Supplemental Figure 2**. Complementarity-determining Region 3 Length prior to and following ^90^Y-TARE. ^90^Y-TARE did not impact the frequency of clonotypes with different complementarity-determining region (CDR3) 3 length. Each dot represents the sum of the clonotypes at the particular CDR3 length for each patient. Blue circles are baseline samples, red circles are post-^90^Y samples.
