# Supplementary material for: Yttrium-90 Induces an Effector Memory Response with Neoantigen Clonotype Expansion: Implications for Immunotherapy
Source: Cancer Res Commun. 2024 Aug 19;4(8):2163–73. doi: 10.1158/2767-9764.CRC-24-0228 (PMC11331567; doi:10.1158/2767-9764.CRC-24-0228)
Supplement: Supplemental tables — 1 to 9 [file crc-24-0228_supplemental_tables_suppst.docx]

**SUPPLEMENTAL MATERIAL**

| **Supplemental Table 1 - Study Cohort Demographics** | |
| --- | --- |
| **Demographic** | **Cohort** |
| **Patients, n (%)** | 94 (100) |
| **Age at HCC diagnosis, years, median (IQR)** | 65 (61 – 67) |
| **Sex, self-reported, male n (%)** | 75 (80) |
| **Race, self-reported, n (%)** |  |
| Caucasian/White | 59 (63) |
| African American/Black | 28 (30) |
| Other | 7 (7) |
| **Cirrhotic etiology, n (%)** |  |
| HCV | 51 (54) |
| SLD | 26 (28) |
| HCV + ALD | 14 (15) |
| Other | 3 (3) |
| **History of Decompensation prior to ^90^Y, n (%)** |  |
| Compensated | 64 (68) |
| Decompensated | 30 (32) |
| **Scores and Staging** |  |
| **ECOG Performance Status, n (%)** |  |
| Score 0 | 66 (74) |
| Score 1 | 23 (26) |
| **Child-Pugh, n (%)** |  |
| A | 67 (71) |
| B | 25 (27) |
| C | 2 (2) |
| **Clinical Labs prior to ^90^Y** |  |
| **Sodium, mM, median (IQR)** | 139 (137 – 141) |
| **Creatinine, mg/dL, median (IQR)** | 0.9 (0.8 – 1.1) |
| **Bilirubin, mg/dL, median (IQR)** | 0.8 (0.6 – 1.4) |
| **Albumin, g/dL, median (IQR)** | 3.5 (3.2 – 3.8) |
| **INR, ratio, median (IQR)** | 1.1 (1.0 – 1.2) |
| **MELD-Na, score (IQR)** | 9 (7 – 10) |
| **MELD 3.0, score (IQR)** | 10 (7 – 12) |
| **HCC Burden** |  |
| **BCLC 2022 Stage, n (%)** |  |
| A | 77 (82) |
| B | 17 (18) |
| **Multifocal, n (%)** |  |
| Solitary | 69 (73) |
| Multifocal | 25 (27) |
| **Index Lesion Diameter, cm, median (IQR)** | 3.5 (2.5 – 4.4) |
| **Cumulative Diameter, cm, median (IQR)** | 3.8 (2.8 – 5.8) |
| **Abbreviations: Interquartile range (IQR), Hepatocellular carcinoma (HCC), Hepatitis C virus (HCV), Steatotic liver disease (SLD), Alcoholic liver disease (ALD), Eastern Cooperative Oncology Group (ECOG), Child-Pugh (CP), Yttrium-90 (^90^Y), International normalized ratio (INR), Model End-Stage Liver Disease (MELD), Barcelona Clinic Liver Cancer (BCLC).** | |

| **Supplemental Table 2. ^90^Y Treatment Characteristics and Response Rates** | |
| --- | --- |
| **^90^Y Therasphere, n (%)** | 94 (100) |
| **Pre-^90^Y Dosimetry** |  |
| **Target Volume, mL, median (IQR)** | 200 (120 – 333) |
| **Target Dose to Volume, Gy, median (IQR)** | 413 (249 – 512) |
| **Lung Shunt Fraction, %, median (IQR)** | 5.3 (3.2 – 8.1) |
| **Post-^90^Y Dosimetry, n (%)** | 57 (61) |
| **Tumor Volume, mL, median (IQR)** | 12.3 (5.2 – 24.6) |
| **Tumor Absorbed Dose, Gy, median (IQR)** | 753 (504 – 1330) |
| **Response to First-Line ^90^Y, n (%)** |  |
| **Target Objective Response Rate** |  |
| OR, n (%) | 76 (84) |
| NOR, n (%) | 14 (16) |
| Response unavailable, n=4 |  |
| **Overall Objective Response Rate** |  |
| OR, n (%) | 64 (70) |
| NOR, n (%) | 27 (30) |
| Response unavailable, n=3 |  |
| Follow-up Time, days, median (IQR) | 75 (42 - 111) |
| **Abbreviations: Interquartile range (IQR), Yttrium-90 (^90^Y), Gray (Gy), Objective response (OR), Nonobjective response (NOR).** | |

| **Supplemental Table 3. Complete Blood Counts Changes Following ^90^Y in Early-Intermediate HCC** | | | |
| --- | --- | --- | --- |
| **Complete Blood Count** | **Baseline** | **Post-^90^Y** | **P value** |
| **White Blood Count, 10^3^/μL, median (IQR)** | 5.1 (3.9 – 7.1) | 4.6 (3.8 – 6.0) | **<0.001** |
| **Granulocyte Count, 10^3^/μL, median (IQR)** | 2.9 (2.1 – 4.2) | 3.3 (2.4 **–** 4.2) | 0.283 |
| **Absolute Lymphocyte Count, 10^3^/μL, median (IQR)** | 1.5 (1.0 – 1.9) | 0.7 (0.5 – 1.1) | **<0.001** |
| **Monocyte Count, 10^3^/μL, median (IQR)** | 0.5 (0.4 – 0.7) | 0.6 (0.4 – 0.7) | 0.081 |
| **Platelet Count, 10^3^/μL, median (IQR)** | 114 (73 – 180) | 110 (78 – 179) | 0.408 |
| **Abbreviations: Yttrium-90 (^90^Y), Hepatocellular carcinoma (HCC), Interquartile range (IQR).** | | | |

| **Supplemental Table 4 - Cirrhosis Etiology and Baseline T Cell Lineage Populations** | | | | | | | | |
| --- | --- | --- | --- | --- | --- | --- | --- | --- |
| **T cell Lineage Populations** | **Cirrhosis Etiology** | | | | | **HCV Viremic Status** | | |
| **CD4^+^ T cells** | **HCV** | **SLD** | **HCV + ALD** | **Other** | **P value** | **Viremic** | **Aviremic** | **P value** |
| T_SCM_, median (IQR) | 29 (16 - 45) | 34 (25 - 52) | 35 (39 - 47) | 33 (19 - 41) | 0.623 | 40 (22 - 47) | 30 (15 - 42) | 0.110 |
| T_CM_, median (IQR) | 94 (91 - 96) | 95 (89 - 97) | 94 (93 - 96) | 88 (83 - 93) | 0.199 | 94 (92 - 96) | 93 (90 - 96) | 0.183 |
| T_EM_, median (IQR) | 2 (1 - 3) | 1 (0.5 - 4) | 3 (0.5 - 5) | 7 (5 - 13) | 0.059 | 2 (1 - 4) | 2 (1 - 4) | 0.772 |
| **CD8^+^ T cells** |  |  |  |  |  |  |  |  |
| T_SCM_, median (IQR) | 16 (10 - 24) | 15 (10 - 29) | 19 (9 - 28) | 8 (7 - 16) | 0.554 | 18 (11 - 24) | 13 (9 - 30) | 0.572 |
| T_CM_, median (IQR) | 75 (65 - 86) | 75 (56 - 84) | 68 (62 - 86) | 61 (30 - 78) | 0.407 | 80 (63 - 87) | 72 (62 - 85) | 0.401 |
| T_EM_, median (IQR) | 18 (10 - 24) | 22 (10 - 36) | 19 (14 - 31) | 37 (13 - 68) | 0.438 | 18 (10 - 26) | 19 (13 - 29) | 0.577 |
| **Abbreviations: Memory stem T cell (T_SCM_), Interquartile range (IQR), Central memory T cell (T_CM_), Effector memory T cells (T_EM_), Hepatitis C virus (HCV), Steatotic liver disease (SLD), Alcoholic liver disease (ALD). T cell lineage are shown as percentage of total CD45RO^+^ or CD45RO^-^. Values are shown as median ± interquartile range.** | | | | | | | | |

| **Supplemental Table 5 - ^90^Y Induced Changes in T cell Phenotypes Based on Overall Response Rate** | | | | | | |
| --- | --- | --- | --- | --- | --- | --- |
|  | **OR** | | **NOR** | | **Matched Pairs** | |
| **T cell Phenotypes** | **Baseline** | **Post-^90^Y** | **Baseline** | **Post-^90^Y** | **Mean Difference** | **Mean Mean** |
| **ALC, median (IQR)** | 1.6 (1.0 – 2.0) | 0.7 (0.5 – 1.2) | 1.4 (0.8 – 1.8) | 0.7 (0.4 – 0.9) | 0.794 | 0.079 |
| **CD4^+^ Subpopulations** |  |  |  |  |  |  |
| **CD4^+^ % of total CD3^+^, median (IQR)** | 40 (30 – 51) | 36 (29 – 54) | 49 (39 – 55) | 41 (29 – 55) | 0.341 | 0.134 |
| **Memory Panel** |  |  |  |  |  |  |
| **Naïve CD4^+^ % of total CD4^+^, median (IQR)** | 49 (41 – 62) | 57 (45 – 68) | 55 (42 – 67) | 62 (56 – 70) | 0.365 | 0.462 |
| **T_SCM_ CD4^+^ % of total CD4^+^, median (IQR)** | 36 (19 – 46) | 28 (16 – 38) | 35 (19 – 48) | 27 (20 – 36) | 0.301 | 0.923 |
| **T_CM_ CD4^+^ % of total CD4^+^, median (IQR)** | 94 (90 – 96) | 93 (88 – 96) | 95 (92 – 96) | 93 (90 – 96) | 0.204 | 0.803 |
| **T_EM_ CD4^+^ % of total CD4^+^, median (IQR)** | 2.0 (0.7 – 5.2) | 2.0 (0.5 – 6.2) | 1.4 (0.6 – 2.7) | 2.5 (0.7 – 4.7) | 0.301 | 0.241 |
| **Senescent Panel** |  |  |  |  |  |  |
| **CD57^+^ CD4^+^ % of total CD4^+^, median (IQR)** | 0.6 (1.1 – 2.0) | 0.6 (0.1 – 2.2) | 0.5 (0.08 – 1.7) | 0.5 (0.06 – 1.1) | 0.512 | 0.446 |
| **KLRG1^+^ CD4^+^ % of total CD4^+^, median (IQR)** | 0.5 (0.2 – 2.4) | 0.7 (0.1 – 2.5) | 0.6 (0.1 – 2.0) | 0.4 (0.02 – 2.7) | 0.809 | 0.363 |
| **Exhaustion Panel** |  |  |  |  |  |  |
| **CTLA4 on total CD4^+^, MFI, median (IQR)** | 186 (181 – 193) | 188 (181 – 198) | 181 (177 – 191) | 191 (186 – 196) | 0.362 | 0.567 |
| **LAG3 on total CD4^+^, MFI, median (IQR)** | 184 (177 – 199) | 186 (178 – 203) | 178 (165 – 187) | 178 (172 – 188) | 0.670 | 0.341 |
| **PD-1 on total CD4^+^, MFI, median (IQR)** | 384 (341 – 409) | 386 (346 – 404) | 383 (353 – 395) | 392 (652 – 403) | 0.957 | 0.678 |
| **CD8^+^ Subpopulations** |  |  |  |  |  |  |
| **CD8^+^ % of total CD3^+^, median (IQR)** | 16 (11 – 22) | 16 (11 – 23) | 16 (12 – 22) | 15 (8.8 – 22) | 0.567 | 0.336 |
| **Memory Panel** |  |  |  |  |  |  |
| **Naïve CD8^+^ % of total CD8^+^, median (IQR)** | 18 (8.6 – 32) | 19 (7.9 – 39) | 18 (11 – 36) | 20 (12 – 34) | 0.361 | 0.961 |
| **T_SCM_ CD8^+^ % of total CD8^+^, median (IQR)** | 14 (9.5 – 25) | 11 (5.4 – 18) | 18 (11 – 31) | 14 (4.9 – 28) | 0.157 | 0.078 |
| **T_CM_ CD8^+^ % of total CD8^+^, median (IQR)** | 73 (62 – 84) | 71 (62 – 79) | 75 (60 – 87) | 69 (56 – 80) | 0.542 | 0.763 |
| **T_EM_ CD8^+^ % of total CD8, median (IQR)** | 18 (12 – 31) | 24 (14 – 33) | 16 (10 – 32) | 20 (17 – 39) | 0.952 | 0.790 |
| **Senescent Panel** |  |  |  |  |  |  |
| **CD57^+^ CD8^+^ % of total CD8^+^, median (IQR)** | 20 (12 – 38) | 22 (10 – 37) | 20 (8.7 – 31) | 24 (11 – 37) | 0.295 | 0.304 |
| **KLRG1^+^ CD8^+^ % of total CD8^+^, median (IQR)** | 19 (11 – 35) | 20 (9 – 35) | 20 (9.0 – 31) | 21 (11 – 34) | 0.354 | 0.323 |
| **Exhaustion Panel** |  |  |  |  |  |  |
| **CTLA4 on total CD8^+^, MFI, median (IQR)** | 171 (166 – 176) | 174 (162 – 179) | 164 (159 – 179) | 171 (164 – 184) | **0.032** | 0.872 |
| **LAG3 on total CD8^+^, MFI, median (IQR)** | 682 (602 – 801) | 741 (641 – 826) | 688 (634 – 736) | 710 (685 – 774) | 0.918 | 0.608 |
| **PD-1 on total CD8^+^, MFI, median (IQR)** | 434 (402 – 470) | 419 (395 – 450) | 456 (404 – 485) | 453 (420 – 487) | 0.276 | **0.042** |
| **Abbreviations: Yttrium-90 (^90^Y), Objective response rate (ORR), Non-objective response rate (NOR), Absolute lymphocyte count (ALC), Interquartile range (IQR), Memory stem T cell (T_SCM_), Central memory T cell (T_CM_), Effector memory T cells (T_EM_), Cytotoxic T-lymphocyte associated protein 4 (CTLA4), Median fluorescence intensity (MFI), Killer lectin-like receptor G1 (KLGR1), Lymphocyte activation gene 3 (LAG3), Programmed cell death-1 (PD-1).** | | | | | | |

| **Supplemental Table 6 – T Cell Receptor Repertoire Changes following ^90^Y** | | | |
| --- | --- | --- | --- |
| **T cell Receptor Parameters** | **Baseline** | **Post-^90^Y** | **P value** |
| Total T cells, median (IQR) | 53,600 (27,000 - 85,700) | 34,900 (19,000 - 50,500) | **<0.001** |
| **TCR Rearrangements** |  |  |  |
| Productive Rearrangements, median (IQR) | 31,000 (17,600 - 51, 500) | 24,800 (14,800 - 35,600) | **0.027** |
| Productive Rearrangements as % of T cells, median (IQR) | 82% (81 - 84) | 82% (81 - 84) |  |
| Out of Frame Rearrangements, median (IQR) | 6,300 (3,700 - 9,700) | 4,700 (2,400 - 6,900) | **0.023** |
| Out of Frame Rearrangements as % of T cells, median (IQR) | 17% (15 - 18) | 17% (15 - 18) |  |
| Stop Rearrangements, median (IQR) | 410 (200 - 580) | 530 (330 - 820) | **0.024** |
| Stop Rearrangements as % of T cells, median (IQR) | 1.4% (1.3 - 1.6) | 1.4% (1.3 - 1.6) |  |
| Unique Clonotypes, median (IQR) | 28,000 (15,000 - 44,000) | 20,000 (11,000 - 32,000) | **0.018** |
| Unique Clonotypes as % of T cells, median (IQR) | 57% (45 - 66) | 58% (45 - 71) | 0.217 |
| **Abbreviations: Yttrium-90 (^90^Y), Interquartile range (IQR), T cell Receptor (TCR).** | | | |

| **Supplemental Table 7. Top 10 Clonotypes in Baseline Based on Presence in Cohort** | | |
| --- | --- | --- |
| **Clonotype (amino acid sequence)** | **Sum Frequency** | **Present in, n (%)** |
| **CASSLGETQYF** | 0.419% | 63 (85) |
| **CASSPSTDTQYF** | 0.278% | 57 (78) |
| **CASSLNTGELFF** | 0.240% | 57 (78) |
| **CASSLGGNTEAFF** | 0.834% | 57 (78) |
| **CASSLGGTDTQYF** | 0.224% | 56 (77) |
| **CASSLEETQYF** | 0.326% | 56 (77) |
| **CASSLQETQYF** | 0.217% | 55 (75) |
| **CASSLNTEAFF** | 0.301% | 55 (75) |
| **CASSLGGNQPQHF** | 1.010% | 55 (75) |
| **CASSLGYEQYF** | 0.442% | 54 (74) |

| **Supplemental Table 8. Top 5 Clonotypes Based on Cirrhosis Etiology at Baseline and Following ^90^Y.** | | |
| --- | --- | --- |
| **Clonotype (amino acid sequence)** | **Sum Frequency** | **Present in, n (%)** |
| **Baseline - Hepatitic C Virus, n=53** |  |  |
| **CASSLGETQYF** | 0.312% | 45 (85) |
| **CASSLNTEAFF** | 0.238% | 43 (81) |
| **CASSLEETQYF** | 0.250% | 43 (81) |
| **CASSLGGNTEAFF** | 0.725% | 42 (79) |
| **CASSLQETQYF** | 0.149% | 41 (77) |
| **Post-^90^Y - Hepatitic C Virus, n=53** |  |  |
| **CASSLGETQYF** | 0.378% | 46 (87) |
| **CASSLGGNTEAFF** | 0.963% | 45 (85) |
| **CASSSSYEQYF** | 0.230% | 38 (72) |
| **CASSLGGTEAFF** | 0.241% | 38 (72) |
| **CASSLGDTQYF** | 0.190% | 38 (72) |
| **Baseline - Steatotic Liver Disease, n=17** |  |  |
| **CASSPSTDTQYF** | 0.072% | 14 (82) |
| **CASSQGYEQYF** | 0.058% | 13 (76) |
| **CASSLNTGELFF** | 0.052% | 13 (76) |
| **CASSLGTGELFF** | 0.067% | 13 (76) |
| **CASSLGTEAFF** | 0.084% | 13 (76) |
| **Post-^90^Y - Steatotic Liver Disease, n=17** |  |  |
| **CASSLGETQYF** | 0.108% | 16 (94) |
| **CASSLTGNTEAFF** | 0.001% | 12 (71) |
| **CASSLGGYEQYF** | 0.077% | 12 (71) |
| **CASSLGGSNQPQHF** | 0.062% | 12 (71) |
| **CASSLGDTQYF** | 0.079% | 12 (71) |

| **Supplemental Table 9. Top 10 Clonotypes Post-^90^Y Based on Presence in Cohort** | | |
| --- | --- | --- |
| **Clonotype (amino acid sequence)** | **Sum Frequency** | **Present in, n (%)** |
| **CASSLGETQYF** | 0.499% | 64 (88) |
| **CASSLGGNTEAFF** | 1.080% | 57 (78) |
| **CASSLGDTQYF** | 0.271% | 51 (70) |
| **CASSLGGTEAFF** | 0.347% | 50 (68) |
| **CASSLQETQYF** | 0.279% | 49 (67) |
| **CASSSSYEQYF** | 0.307% | 48 (66) |
| **CASSLQGNTEAFF** | 0.280% | 48 (66) |
| **CASSLGGYEQYF** | 0.284% | 48 (66) |
| **CASSLTGNTEAFF** | 0.254% | 47 (64) |
| **CASSLSYEQYF** | 0.369% | 47 (64) |
